# Rupture risk parameters upon biomechanical analysis independently change from vessel geometry during abdominal aortic aneurysm growth

**DOI:** 10.1016/j.jvssci.2022.10.004

**Published:** 2022-11-19

**Authors:** David Zschäpitz, Bianca Bohmann, Brigitta Lutz, Hans-Henning Eckstein, Christian Reeps, Lars Maegdefessel, Christian T. Gasser, Albert Busch

**Affiliations:** a Department for Vascular and Endovascular Surgery, Klinikum rechts der Isar, Technical University Munich, Munich, Germany; b Division of Vascular and Endovascular Surgery, Department for Visceral-, Thoracic and Vascular Surgery, Medical Faculty Carl Gustav Carus and University Hospital, Technische Universität Dresden, Dresden, Germany; c Department of Engineering Mechanics, Royal Institute of Technology, Stockholm, Sweden

**Keywords:** Abdominal aortic aneurysm, Aneurysm growth, Finite element method, Rupture risk

## Abstract

**Objective:**

The indication for abdominal aortic aneurysm (AAA) repair is based on a diameter threshold. However, mechanical properties, such as peak wall stress (PWS) and peak wall rupture index (PWRI), influence the individual rupture risk. This study aims to correlate biomechanical and geometrical AAA characteristics during aneurysm growth applying a new linear transformation-based comparison of sequential imaging.

**Methods:**

Patients with AAA with two sequential computed tomography angiographies (CTA) were identified from a single-center aortic database. Patient characteristics included age, gender, and comorbidities. Semiautomated segmentation of CTAs was performed using Endosize (Therenva) for geometric variables (diameter, neck configuration, α/β angle, and vessel tortuosity) and for finite element method A4 Clinics Research Edition (Vascops) for additional variables (intraluminal thrombus [ILT]), vessel volume, PWS, PWRI). Maximum point coordinates from at least one CTA 6 to 24 months before their final were predicted for the final preoperative CTA using linear transformation along fix and validation points to estimate spatial motion. Pearson’s correlation and the *t* test were used for comparison.

**Results:**

Thirty-two eligible patients (median age, 70 years) were included. The annual AAA growth rate was 3.7 mm (interquartile range [IQR], 2.25-5.44; *P* < .001) between CTs. AAA (+17%; *P* < .001) and ILT (+43%; *P* < .001) volume, maximum ILT thickness (+35%; *P* < .001), β angle (+1.96°; *P* = .017) and iliac tortuosity (+0.009; *P* = .012) increased significantly. PWS (+12%; *P* = .0029) and PWRI (+16%; *P* < .001) differed significantly between both CTAs. Both mechanical parameters correlated most significantly with the AAA volume increase (r = 0.68 [*P* < .001] and r = 0.6 [*P* < .001]). Changes in PWS correlated best with the aneurysm neck configuration. The spatial motion of maximum ILT thickness was 14.4 mm (IQR, 7.3-37.2), for PWS 8.4 mm (IQR, 3.8-17.3), and 11.5 mm (IQR, 5.9-31.9) for PWRI. Here, no significant correlation with any of the aforementioned parameters, patient age, or time interval between CTs were observed.

**Conclusions:**

PWS correlates highly significant with vessel volume and aneurysm neck configuration. Spatial motion of maximum ILT thickness, PWS, and PWRI is detectable and predictable and might expose different aneurysm wall segments to maximum stress throughout aneurysm growth. Linear transformation could thus add to patient-specific rupture risk analysis.

**Clinical Relevance:**

Abdominal aortic aneurysm rupture risk assessment is a key feature in future individualized therapy approaches for patients, since more and more data are obtained concluding a heterogeneous disease entity that might not be addressed ideally looking only at diameter enlargement. The approach presented in this pilot study demonstrates the feasibility and importance of measuring peak wall stress and rupture risk indices based on predicted and actual position of maximum stress points including intraluminal thrombus.

**Article Highlights**

• **Type of Research:** Modeling study
• **Key Findings:** Thirty-two patients with abdominal aortic aneurysm AAA with consecutive computed tomography angiographies were semiautomatically segmented for geometrical and biomechanical analysis to identify peak wall stress and rupture index. A linear transformation was used to predict maximum stress and luminal thrombus points during abdominal aortic aneurysm growth and measured their relative position change over the vessel wall.
• **Take Home Message:** The change of positions of maximum intraluminal thrombus thickness, peak wall stress, and peak wall rupture index is independent from most geometric aneurysm measurements during individual aneurysm growth and could thus be relevant for patient-specific rupture risk estimation.

Abdominal aortic aneurysm (AAA) is the most frequent aneurysm disease with the inherent threat of rupture.^1^ Despite very good clinical and patient-related outcomes for elective open or, most frequently, endovascular aortic repair, rupture is still associated with a considerable mortality and postoperative morbidity.^2^

Indications for elective aortic repair are based mostly on reaching a maximum transverse diameter threshold of 50 to 55 mm.^3^^,^^4^ Additionally, fast growth, local symptoms, and eccentric configuration do influence clinical decision-making. However, approximately 1% to 2% of AAAs below the diameter threshold rupture, whereas some huge aneurysms remain intact over a patient’s lifetime.^5^^,^^6^

The aneurysm wall and the underlying intraluminal thrombus (ILT) form a complex biological compartment characterized by cytokine production and the accumulation of neutrophil extracellular traps.^7^ This finding might in part account for the constant aortic remodeling throughout AAA growth along with pathomechanisms inherent to the aneurysm wall.^2^^,^^8^ Semiautomatic postprocessing of computed tomography angiograms (CTA), such as through the finite element method (FEM) has been proposed to study the rupture risk of individual patients. The aneurysm is thought to rupture once the mechanical stress in the vessel wall exceeds the aortic wall strength.^8^^,^^9^ Therefore, either the calculated peak wall stress (PWS) itself, or the maximum between wall stress and an estimated local wall strength, a ratio known as peak wall rupture index (PWRI), serve as rupture risk factors. Additionally, other morphological AAA characteristics, such as vessel volume, ILT, diameter, or vessel length, can be easily and reliably calculated based on segmentation of a patient’s CTA.^10^ Yet, the timely evolution of biomechanical and morphological properties along with aneurysm growth is largely unclear, and their association with eventual rupture remains unknown.^9^^,^^11^

We hypothesized that AAA growth based on diameter enlargement between two consecutive CTAs is accompanied by significant changes in biomechanical and geometrical characteristics. Additionally, we hypothesized that parameters such as PWRI, PWS, and ILT do not simply monotonously grow with aneurysm diameter and that their respective positions also changes with AAA growth. We, therefore, introduce a linear transformation-based comparison of the respective maximum point positions toward the in-depth study of AAA growth dynamics.

## Methods

### Patient identification, inclusion criteria, and data acquisition

**Supplementary Fig 1:**
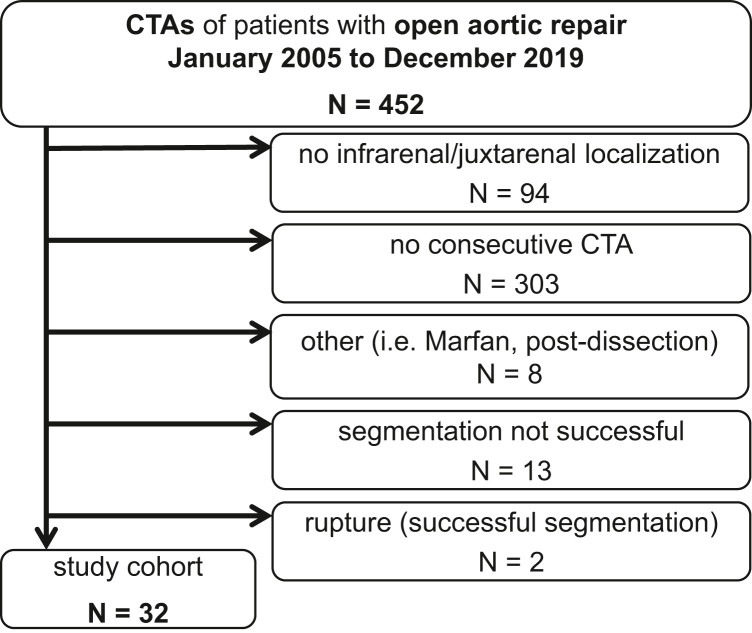
Patient identification flow chart. Most patients were excluded owing to missing consecutive imaging. Two cases of ruptured abdominal aortic aneurysm (AAA) with successful segmentation were excluded since not all fixpoints could be identified for linear transformation. *CTA*, computed tomography angiography.

Patients were retrospectively identified from our aortic database (January 1, 2005, to December 31, 2019) (Supplementary Fig 1). All patients were operated on the infrarenal or juxtarenal aorta (cut-off of >10 mm neck length) for AAA by open surgical means during this time.^3^^,^^10^

Patient data were anonymized for further analysis. The study was performed in accordance with the declaration of Helsinki and approved by the local ethics committee (Ethikkommission Klinikum rechts der Isar: 576/18 S).

We included all patients who had at least one CTA (CT1) 6 to 24 months before their final preoperative CTA (CT2).

Patients with postdissection aneurysms or connective tissue disease were excluded. Also, patients with inadequate CTA data (≥2.5 mm slice thickness; unsuccessful segmentation in VASCOPS or Endosize, as discussed elsewhere in this article) were excluded. Owing to the low number of ruptured AAA cases meeting the inclusion criteria (n = 2, data not shown), ruptures were also excluded.

Data were obtained retrospectively from the department’s aortic database. Patient demographics and comorbidities (age, gender, arterial hypertension, smoking status, peripheral arterial disease, coronary artery disease, hyperlipidemia, diabetes mellitus, chronic obstructive pulmonary disease, renal insufficiency, and obesity) were retrieved from electronic patient records and outpatient follow-up examinations.

### Geometric AAA analysis

The morphological analysis was performed semiautomatically with Endosize (Therenva, Rennes, France), a software for clinical assessment of AAAs as well as for endovascular aortic repair planning (www.therenva.com/endosize) as previously described and validated by us and others.^10^^,^^12^^,^^13^ Briefly, defined setpoints were manually entered in the segmented CTA (all noncardiac gated). Then, a centerline was calculated and verified with eventual manual adjustment. Calculated parameters included: suprarenal to infrarenal neck angulation (α), infrarenal neck to AAA angulation (β), maximum transverse diameter, neck length, proximal and distal neck diameter (lowest renal artery to the beginning of the aneurysm), and aortic/iliac tortuosity index (centerline to direct raceline distance ratio: lowest renal artery to aortic bifurcation/aortic bifurcation to inguinal ligament).^14^

Additionally, the maximum AAA diameter was calculated and verified by classic means, with the outer edge transversal measurement of the maximum diameter in a three-dimensional multiplanar reconstruction.

### Biomechanic AAA analysis

A semiautomated biomechanics FEM analysis was performed using A4clinics Research Edition (Vascops GmbH, Graz, Austria) as described previously.^8^^,^^11^ Briefly, a three-dimensional model of the AAA is semiautomatically segmented from CTA images, identifying the lumen, the ILT, and the outer contour of the vessel wall. The segmentation covers the aortic segment between the lowest renal artery and the aortic bifurcation, and the investigator manually corrects the model in line with the instructions for use, that is, if given the segmentation mismatch exceeds 2 mm. A standardized arterial pressure of 140/80 mm Hg was used for all FEM computations, and model outputs are the total vessel volume, maximal luminal diameter, lumen volume, maximal ILT thickness, ILT volume, mean ILT stress, PWS, and PWRI. The PWS represents the maximal stress, and the PWRI is the maximum ratio between wall stress and wall strength in the aneurysm.

### Linear transformation analysis

Given the coordinates (*x*, *y*, *z*) of the points of maximum ILT thickness, PWS, and PWRI in CT1, linear transformation (also known as rigid registration or affine transformation) was used to predict said points in CT2. Minimizing the error through least square optimization of the *x*, *y*, and *z* coordinates of up to nine corresponding points (left and right renal arteries, superior mesenteric artery, aortic bifurcation, proximal left and right common iliac arteries, and one to three lumbar arteries if available) in CT1 and CT2 determined the transformation matrix (MATHEMATICA 12.0, Wolfram, Champaign, IL). An additional point (ie, calcified plaque or inferior mesenteric artery) clearly visible in both CTAs validated the transformation matrix. The applied linear transformation was considered successful if the distance between the predicted position and the actual position of the validation point in CT2 was less than 15 mm.

All measurements, including biomechanical parameters and the linear transformation, were performed by an experienced analyzer (D.Z.) and reviewed by an experienced vascular surgeon and analyzer (A.B., T.C.G.). Upon discrepant results, all three investigators performed a joint analysis.

### Statistics and figure composition

Patients and AAA characteristics are shown as median with interquartile range (IQR) for continuous variables and absolute numbers with percentages for categorical data.

Given the small number of patients, a Wilcoxon test was used to test for significant changes between CT1 and CT2 as well as between the different groups. It considers different variances across the compared groups and minimizes the possible influence of outliers. Pearson correlation coefficient (r) tested the linear correlations between different variables, and the level of significance was set at a *P* value of less than .05. All statistical analyses were performed using R version 4.0.3 (R Foundation for Statistical Computing, Vienna, Austria; https://www.r-project.org/) and graphics were created using the ggplot2 package.

## Results

In total, 32 patients met the inclusion criteria and were included in the study (Supplementary Fig 1). Thirty patients were male (median age, 70 years; IQR, 62-75 years). Detailed patient characteristics including comorbidities are listed in Table I. Two patients had symptomatic AAA before operation and seven patients had additional iliac aneurysms.

**Table I:** Patient (n = 32) and aneurysm characteristics

Characteristics	No. (%)
Age at operation, years	70 [62-75]^a^
Male sex	30 (94)
Comorbidities	
Hypertension	26 (81)
Diabetes	9 (28)
Hyperlipidemia	24 (75)
Heart disease	13 (41)
COPD	7 (22)
PAOD	5 (16)
Smoking status	
Current	20 (63)
Ex	7 (22)
Never	3 (9)
Medication	
ASA/clopidogrel	20 (63)
ACE inhibitors	10 (31)
Statins	18 (56)
Metformin	2 (6)
Insulin	1 (3)
Elevated/reduced serum parameters	
C-reactive protein ≥0.5 mg/dL	8 (44)
Leukocytes <3.5/>9.5 × 10³/μL	3 (9)
Thrombocytes <80/>350 × 10³/μL	1 (3)
Creatinine >1.2 mg/dL	5 (16)
AAA characteristics	
Asymptomatic (vs symptomatic)	30 (94)
Localization	
Infrarenal (vs juxtarenal)	19 (59)
Plus iliac	7 (22)

*ACE*, Angiotensin converting enzyme; *ASA,* aspirin; *COPD*, chronic obstructive pulmonary disease; *PAOD*, peripheral arterial occlusive disease.

A symptomatic AAA is two times abdominal pain without other causes; an infrarenal vs juxtarenal AAA is >10 mm neck length; a plus iliac aneurysm is two times bilateral.

a Median [interquartile range].

**Fig 1:**
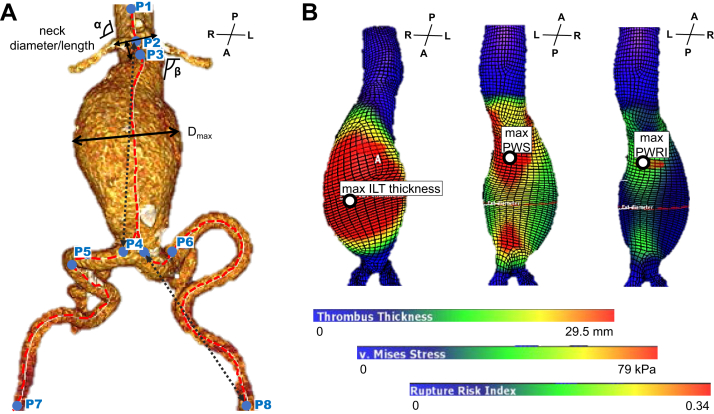
Endosize and Vascops data acquisition. The pictures display an exemplary three-dimensional computed tomography angiography (CTA) reconstruction after successful semiautomated segmentation. **(A)** Using Endosize, the neck diameter and length as well as α and β angulations are calculated. The maximum abdominal aortic aneurysm (AAA) diameter (D_max_) is calculated perpendicular to the center line (red dotted line). The aortic/iliac tortuosity indices are calculated as the ratio centerline/raceline (black dotted arrow) between P2 (lowest renal artery)/P4 (aortic bifurcation) and P4/P8 (inguinal ligament), respectively. **(B)** Using Vascops A4 Clinics Research, the finite element method (FEM) captures areas (displayed as heatmap) and maximum points of intraluminal thrombus (*ILT*, mm) thickness, peak wall stress (*PWS*; v. Mises stress, kPa) and peak wall rupture index (*PWRI*; rupture risk index). Orientation of reconstruction represented by anterior (*A*), posterior (*P*), left (*L*), and right (*R*).

The time difference between the two analyzed CTAs was 14 months (IQR, 9-24 months) (Table II), within which the AAA diameter increased significantly by 3.7 mm/year (IQR, 2.25-5.44 mm/year) (absolute values, CT1: 50 mm [IQR, 45.8-52.0 mm]; to CT2: 55 mm [IQR, 52.0-56.8 mm]; *P* < .001). Upon morphological analysis, only the β angle (+1.96°; *P* = .017) and the iliac tortuosity index (+0.009; *P* = .012) changed significantly; all other parameters showed only slight alterations (Fig 1, *A*, Table II). In contrast, the volumes of the entire aneurysm (+17%; *P* < .001) and the ILT (+43%; *P* < .001), as well as the maximum ILT thickness (+35%; *P* < .001) increased significantly. Also, the changes of PWS (+12%; *P* = .0029) and PWRI (+16%; *P* < .001) were significant from CT1 to CT2 (Fig 1, *B*, Table II).

**Table II:** Sequential Endosize and Vascops computed tomography (*CT*) analysis data

	CT1	CT2	Δ (absolute)	Δ (%)	*P* value (Wilcoxon)	Normalized per 12 months
Time, months			14 [9 to 24]			
AAA diameter, mm	49.9 [45.8 to 52.0]	55.0 [52.0 to 56.8]	5.4 [3.1 to 7.4]	11 [6 to 16]	**.0000018**	3.70 [2.25 to 5.44]
α angle (°)	14.8 [10.9 to 20.7]	18.2 [10.8 to 24.7]	0.5 [–1.5 to 6.2]	2.4 [–13.0 to 48.0]	.23	0.38 [–0.98 to 2.82]
β angle (°)	28.7 [21.5 to 33.7]	30.3 [22.6 to 40.0]	2.9 [–1.8 to 5.9]	10 [–4 to 18]	**.017**	1.96 [–0.98 to 3.17]
Neck length, mm	20.5 [9.0 to 37.5]	20.0 [7.0 to 41.8]	–0.5 [–4.5 to 3.8]	–1 [–23 to 20]	.79	–0.20 [–3.71 to 1.60]
Neck diameter, mm	24 [22.0 to 27.68	23.9 [22.6 to 26.0]	–0.9 [–1.8 to 1.0]	–4 [–8 to 4]	.13	–0.41 [–1.25 to 0.54]
CIA length left	61 [45 to 76]	57 [49 to 69]	–2 [–10 to 4]	–4 [–16 to 8]	.065	–1.72 [–7.03 to 2.42]
CIA length right	59 [40 to 69]	54 [42 to 69]	–2 [–5 to 3]	–3 [–10 to 5]	.22	–0.60 [–5.14 to 2.68]
Aortic tortuosity index	1.07 [1.06 to 1.12]	1.08 [1.06 to 1.14]	0.002 [–0.006 to 0.022]	0.2 [–0.4 to 2.1]	.22	0.002 [–0.004 to 0.012]
Iliac tortuosity index	1.30 [1.21 to 1.36]	1.30 [1.23 to 1.38]	0.016 [–0.012 to 0.045]	1.3 [–1.0 to 3.5]	**.012**	0.009 [–0.017 to 0.026]
Maximum lumen diameter, mm	37.0 [32.4 to 41.6]	39.5 [34.9 to 46.5]	3.9 [1.1 to 7.1]	13 [4 to 20]	**.000032**	3.26 [0.93 to 4.67]
Maximum ILT thickness, mm	14.0 [9.0 to 20.0]	17.7 [14.9 to 27.0]	4.8 [1.1 to 7.2]	35 [5 to 61]	**.000032**	3.04 [1.36 to 4.96]
Total lumen volume, mm^3^	68 [48 to 82]	71 [54 to 95]	8 [3 to 18]	18 [4 to 40]	**.00028**	6.70 [1.85 to 11.50]
Total vessel volume, mm^3^	129 [93 to 164]	150 [121 to 206]	24 [18 to 45]	17 [13 to 38]	**.0000012**	20.85 [14.77 to 30.70]
Total ILT volume, mm^3^	37 [26 to 64]	63 [40 to 92]	18 [7 to 32]	43 [16 to 81]	**.000044**	13.20 [5.56 to 25.66]
PWS, kPa	190 [156 to 229]	195 [178 to 257]	24 [2 to 38]	12 [1 to 23]	**.0029**	12.66 [1.41 to 26.65]
PWRI	0.36 [0.33 to 0.42]	0.38 [0.31 to 0.53]	0.05 [0.00 to 0.10]	16 [0 to 24]	**.00051**	0.03 [0.00 to 0.07]

*AAA*, Abdominal aortic aneurysm; *CIA*, common iliac artery; *ILT*, intraluminal thrombus; *PWRI*, peak wall rupture index; *PWS*, peak wall stress.

Values are as median [interquartile range].

Boldface entries indicate statistical significance (Wilcoxon rank–sum test).

**Supplementary Fig 2:**
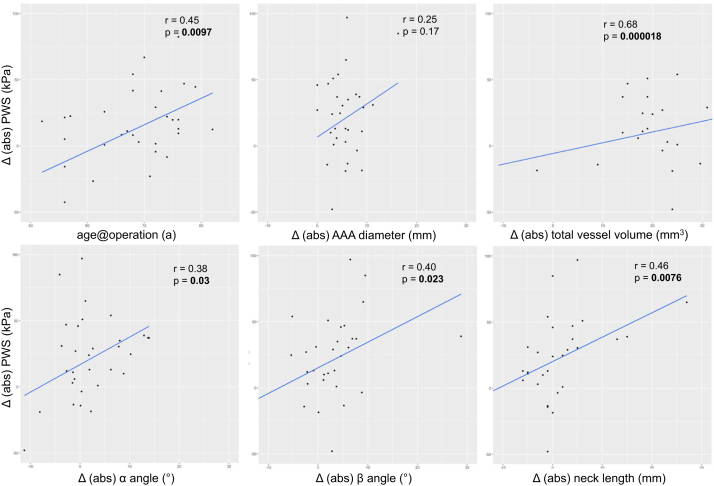
Pearson correlation plots for Δ peak wall stress (*PWS*). Correlation coefficient and respective *P* values are displayed for six exemplary correlation plots using absolute difference between at least one CTA (CT1) 6-24 months before their final preoperative CTA (CT2). A *P* value of less than .05 is considered significant and highlighted bold; all values are shown in Table III.

**Supplementary Fig 3:**
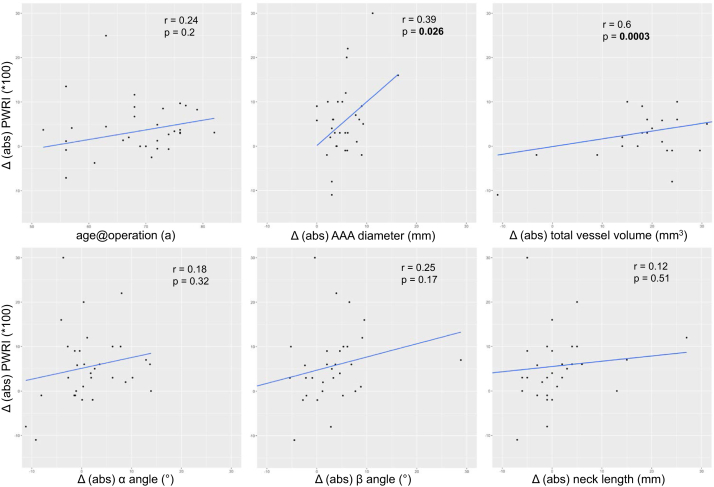
Pearson correlation plots for Δ peak wall rupture index (*PWRI*). Correlation coefficient and respective *P* values are displayed for six exemplary correlation plots using absolute difference between at least one CTA (CT1) 6-24 months before their final and the final preoperative CTA (CT2). A *P* value of less than .05 is considered significant and highlighted bold; all values are shown in Table III.

These changes in PWS and PWRI correlated most significantly with the total AAA volume increase (PWS: correlation coefficient r = 0.68 [*P* < .001]; PWRI: r = 0.6 [*P* < .001]) (Supplementary Fig 2, Table III). Only the difference in PWRI showed a weak correlation with aneurysm diameter increase (r = 0.39; *P* = .026) (Supplementary Fig 3). Changes in PWS correlated best with the configuration of the aneurysm neck. Additionally, a weak correlation with patient age was noted (r = 0.45; *P* = .010). Naturally, most values correlated well with the time interval between CT scans (Table II, Supplementary Table I).

**Table III:** Correlation analysis of absolute peak wall stress (*PWS*) and peak wall rupture index (*PWRI*) changes with age, geometric and volumetric abdominal aortic aneurysm (*AAA*) changes

Pearson correlation	Δ (abs) PWS		Δ (abs) PWRI	
	r	*P* value	r	*P* value
Δ age at operation	0.45	**.0097**	0.24	.20
Δ (abs) AAA diameter	0.25	.17	0.39	**.026**
Δ (abs) α angle	0.38	**.03**	0.18	.32
Δ (abs) β angle	0.40	**.023**	0.25	.17
Δ (abs) neck length	0.46	**.0076**	0.12	.51
Δ (abs) neck diameter	0.095	.61	0.12	.51
Δ (abs) CIA length left	–0.015	.93	0.091	.62
Δ (abs) CIA length right	0.0061	.97	0.11	.5
Δ (abs) aortic tortuosity index	0.19	.31	0.22	.23
Δ (abs) iliac tortuosity index	0.21	.24	0.12	.51
Δ (abs) maximum lumen diameter	0.61	**.00021**	0.47	**.0063**
Δ (abs) maximum ILT thickness	0.30	.092	0.30	.1
Δ (abs) total lumen volume	0.64	**.00007**	0.45	**.01**
Δ (abs) total vessel volume	0.68	**.000018**	0.60	**.00032**
Δ (abs) total ILT volume	0.25	.16	0.33	.062

*CIA*, Common iliac artery; *ILT*, intraluminal thrombus.

Absolute values are correlated. Boldface entries indicate statistical significance.

Examples of correlation plots are displayed in Supplementary Figs 2 and 3).

**Fig 2:**
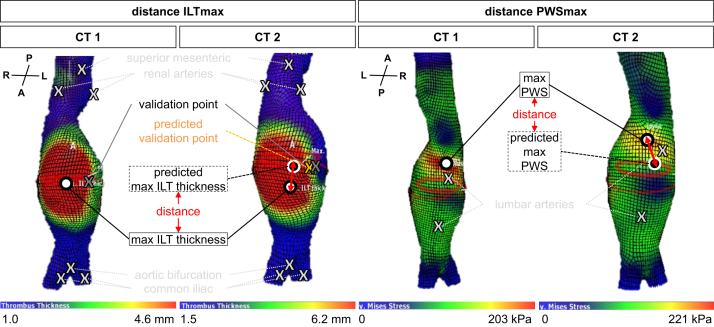
Linear transformation and maximum point motion assessment for maximum intraluminal thrombus (*ILT*) thickness and peak wall stress (*PWS*). Using Vascops, several fixpoints close to, but not within, the actual aneurysm were defined and the *x*, *y*, and *z* coordinates extracted (ie, aortic bifurcation, superior mesenteric, renal, lumbar and iliac arteries: grey X). These defined the matrix for linear transformation and prediction of the validation point (ie, inferior mesenteric artery: orange X) and the points of maximum ILT, PWS and PWRI (black/white dot, s.) (see Supplementary Fig 4 for the PWRI). The distance between the actual and the predicted validation point in CT2 was supposed to be less than 15 mm for study inclusion. Then the distances between actual and predicted maximum points were calculated. Orientation of reconstruction represented by anterior (*A*), posterior (*P*), left (*L*), and right (*R*).

**Supplementary Fig 4:**
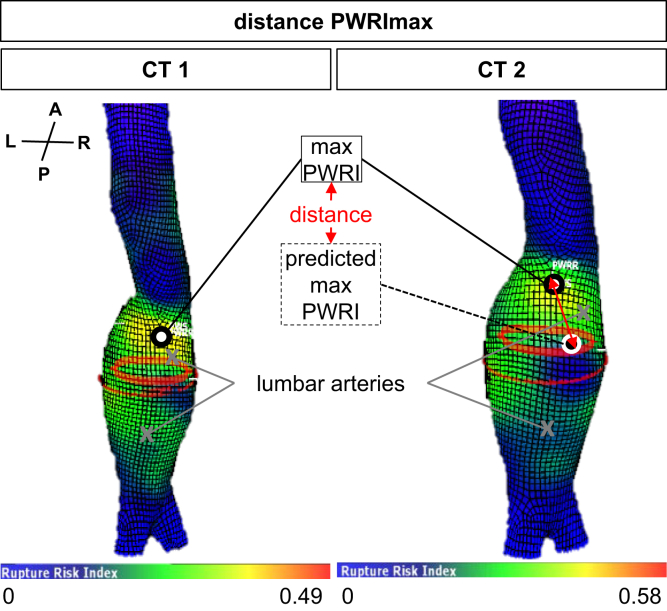
Linear transformation and maximum point motion assessment for peak wall rupture index (*PWRI*). Using Vascops, several fixpoints, close to, but not within the actual aneurysm were defined and the *x*, *y*, and *z* coordinates extracted (ie, aortic bifurcation, superior mesenteric, renal, lumbar and iliac arteries: grey X). These defined the matrix for linear transformation and prediction of the validation point (ie, inferior mesenteric artery: orange X) and the points of maximum intraluminal thrombus (ILT), peak wall stress (PWS) and PWRI (black/white dot, see Fig 2 for the maximum ILT thickness and PWS). The distance between the actual and the predicted validation point in the final preoperative CTA (CT2) was supposed to be less than 15 mm for study inclusion. Then the distances between actual and predicted maximum points were calculated. Orientation of reconstruction represented by anterior (*A*), posterior (*P*), left (*L*), and right (*R*.)

**Supplementary Fig 5:**
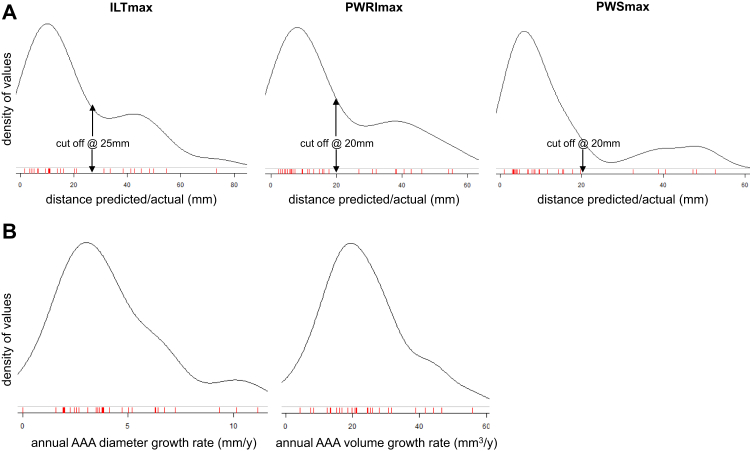
Density plot analysis. **(A)** The density of values for the distance between actual and predicted maximum intraluminal thrombus (*ILTmax*), peak wall rupture index (*PWRImax*), and peak wall stress (*PWSmax*) point and **(B)** annual diameter and volume growth rate are shown. A cut-off for group comparison was identified based on the curve shape. Values for comparative analysis are displayed in Supplementary Table II.

For additional analyses, the spatial motion of the point at which maximum ILT thickness, PWS, and PWRI appeared was monitored. To this end, said points were projected from CT1 to CT2 using the aforementioned linear transformation, enabling us to measure the distance to the positions, where maximum ILT thickness, PWS, and PWRI actually appeared in CT2 (Fig 2, Supplementary Fig 4). These distances were 14.4 mm (IQR, 7.3-37.2 mm) for the maximum ILT, 8.4 mm (IQR, 3.8-17.3 mm) for the maximum PWS, and 11.5 mm (IQR, 5.9-31.9 mm) for the maximum PWRI. The distance between the predicted and the literal positions of the validation point was 7.9 mm (IQR, 5.3-10.9 mm) (data not shown). However, no significant correlations of these motions with any of the morphological or biomechanical parameters, patient age, or time interval of CTAs were observed (Table IV). Additionally, rank-sum tests for differences between patients with large and small motions between the three maximum points did not reveal any significant differences in those groups (Supplementary Fig 5, *A*; Supplementary Table II). The annual AAA growth rate and total volume growth rate were equally distributed and thus not further analyzed in groups (Supplementary Fig 5, *B*).

**Table IV:** Correlation of maximum intraluminal thrombus (*ILT*) thickness, peak wall stress (*PWS*) and peak wall rupture index (*PWRI*) spatial distance changes with geometric, volumetric and biomechanical parameters

Pearson correlation	Distance max ILT		Distance PWRI		Distance PWS	
	R	*P* value	r	*P* value	r	*P* value
Δ (abs) age at operation	–0.15	.41	–0.0823	.66	0.0806	.66
Δ (abs) AAA diameter	0.15	.41	0.205	.26	0.0571	.76
Δ (abs) α angle	–0.35	.053	0.0679	.71	0.215	.24
Δ (abs) β angle	–0.070	.70	0.186	.31	0.188	.30
Δ (abs) neck length	–0.075	.68	0.104	.57	0.155	.4
Δ (abs) neck diameter	–0.26	.15	–0.0468	.80	–0.206	.27
Δ (abs) aortic tortuosity index	0.34	.058	0.144	.43	0.319	.075
Δ (abs) iliac tortuosity index	–0.043	.81	0.0309	.87	0.234	.2
Δ (abs) maximum lumen diameter	0.086	.64	0.148	.42	0.110	.55
Δ (abs) maximum ILT thickness	0.18	.32	–0.330	.065	–0.0659	.72
Δ (abs) total lumen volume	–0.062	.74	0.113	.54	0.110	.55
Δ (abs) total vessel volume	0.020	.91	–0.154	.40	–0.143	.43
Δ (abs) total ILT volume	0.17	.35	–0.208	.25	–0.206	.26
Δ (abs) PWS	0.064	.73	0.0421	.82	–0.106	.56
Δ (abs) PWRI	0.17	.34	–0.124	.5	–0.0132	.94

*AAA*, Abdominal aortic aneurysm.

Absolute values are correlated. Boldface entries indicate statistical significance.

## Discussion

To the best of our knowledge, this study explored for the first time the spatial motion of characteristic geometrical and biomechanical points of the aortic wall during AAA growth. A linear transformation has been used to explore said positions during consecutive aortic imaging. Our pilot study suggests that the motion of the point where extreme geometrical and biomechanical parameters have been identified is independent from other growth parameters, especially AAA diameter. Additionally, we demonstrate that an increase in maximum PWS and PWRI correlates highly significant with AAA volume and neck configuration.

Between CT1 and CT2, the aneurysm diameter changed significantly, along aneurysm volume and ILT characteristics (Table II). AAA volume was demonstrated to grow independently and faster than diameter in the past.^15^ Recent research suggests that volume growth, specifically the ratio between lumen and thrombus volume, might be a more sensitive parameter for eventual symptomatic state of disease or even rupture.^11^^,^^16^^,^^17^ Therefore, AAA volume has even been included in medical intervention studies on AAA growth inhibition.^18^ Accordingly, our results demonstrate a highly significant positive correlation of changes in PWS and PWRI with total vessel, luminal, and ILT volume (Table III, Supplementary Figs 2 and 3).^19^

In particular, the rapid growth of ILT volume (43%) and thickness (35%) in comparison with AAA diameter (11%) might be an underestimated pathologic feature (Table I).^20^ The ILT is considered not only as a viscoelastic structural component with beneficial stress-buffering properties, but also as an enzymatically active compartment producing cytokines and adding to the constant remodeling of the aortic wall.^2^^,^^8^^,^^21^ Interestingly, the spatial motion of the point of the maximum ILT thickness, in comparison with the positions of maximum PWS and PWRI, was the most pronounced in our study (Fig 2, Supplementary Table II). Additionally, patients with greater distances between predicted and the literal position of maximum ILT thickness showed a significantly increased PWRI (Supplementary Table II).^22^ A large movement of the point of the maximum ILT thickness during aneurysm growth might, therefore, be linked to an increase in risk of rupture based on previous similar speculations.^9^^,^^21^^,^^23^

AAA rupture is a local event in the aortic wall, and a large movement in the position of PWS or PWRI constantly exposes a new segment of the vessel wall to risk. To cope with that, the aneurysm wall remodels accordingly and previous histologic comparisons have demonstrated a distinct morphology in AAA wall samples with high versus low PWRI.^24^ However, histologic appearance is very heterogeneous among patient samples and the morphological influence of ILT thickness is unclear.^25^^,^^26^ Thus, new imaging methods using radioactive or molecular magnetic resonance imaging probes are currently evaluated on a preclinical level to combine histologic features of remodeling with in vivo imaging approaches.^27^, ^28^, ^29^ Ideally, such data will be considered in future versions of FEM-based AAA biomechanics to integrate remodeling during aneurysm growth and increase the precision of the rupture risk assessment.

This pilot study introduced a fundamentally new approach with several limitations, however. Only a small number of patients could be included in the study, mostly owing to missing consecutive imaging (Supplementary Fig 1). The majority of patients were male (94%; all Caucasian); however, the significance sex and race disparities were unclear on FEM analyses (Table I).^9^ Considering the high heterogeneity among patients with AAA, this might conceal possible errors during statistical analysis. Ideally, the method should be validated including patients with more than CTAs. Semiautomated CTA segmentation with consecutive diameter calculation harbors the risk of false measurements, if not reviewed and manually corrected as needed.

Although several groups have demonstrated the feasibility and applicability for different research purposes, morphological and FEM analyses are technically demanding and the CTAs included are not standardized (ie, no cardiac gating).^10^^,^^11^^,^^13^ In addition, using patient-specific versus standardized blood pressures, as done in our FEM-based biomechanical analysis, is a matter of current debate, probably also in the context of gender differences.^8^^,^^9^ However, in contrast with the values of PWS and PWRI, their position, and the position of the maximum ILT, is insensitive to blood pressure.

Larger studies with more patients and possibly additional consecutive imaging with more than two time points are needed to better evaluate the method and results presented here. More crucially, analyses of ruptured AAA cases with consecutive preceding aortic imaging are scarce.^30^ Ultimately, studies with prospective patient analyses are needed to compare the patient-individual rupture risk alongside standard diameter evaluation as the current gold standard for preemptive AAA repair to gain future clinical perspective.^5^

## Conclusions

Increased PWS correlated highly significantly with vessel volume and aneurysm neck configuration, whereas an increased PWRI correlated with vessel volume and AAA diameter. In addition, the motion of the maximum ILT thickness, PWS, and PWRI positions is independent from most geometric aneurysm measurements during aneurysm growth. It might therefore bear additional valuable information to assess AAA rupture risk, because there is a constant exposure of different aortic segments to differential PWS.

## Author Contributions

Conception and design: TCG, AB

Analysis and interpretation: DZ, LM, CR, TCG, AB

Data collection: DZ, BB, BL, TCG

Writing the article: DZ, BL, TCG, AB

Critical revision of the article: DZ, BB, BL, LM, HHE

Final approval of the article: DZ, BB, BL, LM, CR, HHE, TCG, AB

Statistical analysis: BB

Obtained funding: LM, HHE, AB

Overall responsibility: AB

## Figures and Tables

**Supplementary Table I:** Correlation of analyzed values with time

Pearson correlation	Δ (abs) time	
	r	*P* value
Δ (abs) age at operation	0.048	.795
Δ (%) AAA diameter	0.57	**.000735**
Δ (%) α angle	0.15	.417
Δ (%) β angle	0.47	**.00636**
Δ (%) neck length	0.066	.719
Δ (%) neck diameter	–0.024	.896
Δ (%) aortic tortuosity index	0.18	.327
Δ (%) iliac tortuosity index	0.36	**.0461**
Δ (%) maximum lumen diameter	0.41	**.0197**
Δ (%) maximum ILT thickness	0.61	**.00024**
Δ (%) total lumen volume	0.24	.194
Δ (%) total vessel volume	0.56	**.000835**
Δ (%) total ILT volume	0.54	**.00135**
Δ (%) PWS	0.52	**.00236**
Δ (%) PWRI	0.42	**.0179**
Distance max ILT	–0.12	.506
Distance PWS	–0.14	.455
Distance PWRI	0.15	.419

*AAA*, Abdominal aortic aneurysm; *ILT*, intraluminal thrombus; *PWRI*, peak wall rupture index; *PWS*, peak wall stress.

Percentage change is correlated with the time interval (correlation coefficient r). Boldface entries indicate statistical significance.

**Supplementary Table II:** Group comparison high and low intraluminal thrombus (*ILT*) thickness, peak wall stress (*PWS*) and peak wall rupture index (*PWRI*) spatial distance

	Maximum ILT		
	Small distance	Large distance	*P* value (Wilcoxon)
Maximum ILT (cut-off 25 mm: 22 vs 10)			
Δ (abs) age (years)	69.5 [61.5 to 73.5]	70.5 [64.3 to 75.8]	.744
Δ (abs) AAA diameter, mm	5.4 [3.4 to 6.2]	5.1 [3.1 to 9.2]	.515
Δ (abs) α angle (°)	1.5 [1.1 to 7.4]	–0.5 [–3.5 to 2.1]	.204
Δ (abs) β angle (°)	3.5 [–1.8 to 5.9]	2.0 [0.0 to 4.7]	.760
Δ (abs) neck length, mm	–0.5 [–3.0 to 2.0]	–0.5 [–4.3 to 3.8]	.968
Δ (abs) neck diameter, mm	–0.2 [–1.6 to 1.3]	–1.7 [–2.1 to –0.6]	.133
Δ (abs) CIA length left, mm	–2.0 [–5.0 to 1.8]	–1.0 [–5.3 to 4.8]	.527
Δ (abs) CIA length right, mm	–4.0 [–10.8 to 2.5]	–1.0 [–4.0 to 4.0]	.360
Δ (abs) aortic tortuosity index	0.0005 [–0.0056 to 0.0108]	0.0090 [–0.0034 to 0.0492]	.458
Δ (abs) iliac tortuosity index	0.0147 [–0.0098 to 0.0428]	0.0164 [–0.0069 to 0.0375]	.952
Δ (abs) maximum lumen diameter, mm	3.4 [1.1 to 6.3]	6.4 [1.6 to 7.4]	.350
Δ (abs) maximum ILT thickness, mm	5.5 [1.2 to 6.4]	3 [1.6 to 7.0]	.857
Δ (abs) total lumen volume, mm^3^	6.8 [3 to 14]	11 [5 to 22]	.434
Δ (abs) total vessel volume, mm^3^	24 [19 to 45]	22 [16 to 36]	.807
Δ (abs) total ILT volume, mm^3^	18 [12 to 27]	21 [3 to 38]	.826
Δ (abs) PWS, kPa	13.0 [2.4 to 33.9]	39.0 [15.5 to 53.3]	.064
Δ (abs) PWRI	0.030 [–0.008 to 0.060]	0.095 [0.053 to 0.145]	**.046**
PWRI (cut-off 20 mm: 22 vs 10)			
Δ (abs) age (years)	70 [61.5 to 75.5]	69 [63.8 to 73.5]	.744
Δ (abs) AAA diameter, mm	3.0 [3.0 to 5.8]	7.1 [4.5 to 8.9]	**.036**
Δ (abs) α angle (°)	–1.4 [–1.4 to 5.6]	0.4 [–1.4 to 6.7]	.900
Δ (abs) β angle (°)	–2.4 [–2.4 to 5.1]	3.5 [2.2 to 6.5]	.173
Δ (abs) neck length, mm	–3.0 [–3.0 to 2.0]	0.0 [–4.0 to 3.8]	.744
Δ (abs) neck diameter, mm	–1.9 [–1.9 to 1.0]	–1.3 [–1.8 to 0.1]	.704
Δ (abs) CIA length left, mm	–5.8 [–5.8 to 1.0]	0.0 [–4.5 to 3.0]	.610
Δ (abs) CIA length right, mm	–8.0 [–8.0 to 4.0]	–2.5 [–12.3 to 2.3]	.767
Δ (abs) aortic tortuosity index	–0.0056 [–0.0056 to 0.011]	0.0121 [–0.0032 to 0.0345]	.388
Δ (abs) iliac tortuosity index	–0.0083 [–0.0083 to 0.041]	0.0190 [–0.013 to 0.0518]	.984
Δ (abs) maximum lumen diameter, mm	3.4 [1.2 to 5.5]	6.7 [1.7 to 7.2]	.452
Δ (abs) maximum ILT thickness, mm	5.5 [1.5 to 8.8]	4.0 [0.9 to 5.6]	.269
Δ (abs) total lumen volume, mm^3^	4 [4 to 14]	5 [1 to 22]	.795
Δ (abs) total vessel volume, mm^3^	17 [17 to 51]	23 [19 to 31]	.823
Δ (abs) total ILT volume, mm^3^	7 [7 to 34]	21 [15 to 25]	.795
Δ (abs) PWS, kPa	18.5 [3.7 to 44.2]	30.0 [0.1 to 36.5]	.984
Δ (abs) PWRI	0.035 [0.005 to 0.098]	0.055 [0.003 to 0.083]	.871
PWS (cut off 20 mm: 26 vs 6)			
Δ (abs) age (years)	69.5 [61.5 to 73.8]	70.5 [66.3 to 75.5]	.717
Δ (abs) AAA diameter, mm	3.1 [3.1 to 6.2]	5.2 [3.5 to 7.4]	.828
Δ (abs) α angle (°)	–1.5 [–1.5 to 3.4]	3.4 [–0.9 to 11.2]	.494
Δ (abs) β angle (°)	–0.8 [–0.8 to 5.1]	3.6 [–2.9 to 6.5]	.847
Δ (abs) neck length, mm	–4.5 [–4.5 to 2.0]	1.5 [–1.0 to 5.5]	.410
Δ (abs) neck diameter, mm	3.1 [3.1 to 6.2]	5.2 [3.5 to 7.4]	.828
Δ (abs) CIA length left, mm	–5.0 [–5.0 to 2.8]	–2.5 [–5.3 to 1.8]	.771
Δ (abs) CIA length right, mm	–7.8 [–7.8 to 4.0]	–6.0 [(–15.0) to (–4.0)]	.080
Δ (abs) aortic tortuosity index	–0.0008 [–0.0056 to 0.011]	0.0268 [0.0102 to 0.0627]	.062
Δ (abs) iliac tortuosity index	0.0153 [–0.0098 to 0.032]	0.0594 [–0.0026 to 0.1032]	.264
Δ (abs) maximum lumen diameter, mm	0.9 [0.9 to 7.1]	6.1 [4.8 to 6.8]	.299
Δ (abs) maximum ILT thickness, mm	1.8 [1.8 to 6.4]	3.4 [–0.6 to 7.2]	.655
Δ (abs) total lumen volume, mm^3^	3 [3 to 15]	8 [4 to 20]	.944
Δ (abs) total vessel volume, mm^3^	18 [18 to 45]	22 [19 to 28]	.735
Δ (abs) total ILT volume, mm^3^	19 [19 to 32]	16 [–3 to 20]	.408
Δ (abs) PWS, kPa	3.7 [3.7 to 34.0]	38.0 [–0.8 to 48.0]	.515
Δ (abs) PWRI	0.003 [0.003 to 0.098]	0.060 [0.008 to 0.068]	.885

*AAA*, Abdominal aortic aneurysm; *CIA*, common iliac artery.

Values are median [interquartile range].

Boldface entries indicate statistical significance (Wilcoxon-rank sum test).

The distribution of values and respective cut-off are displayed in Supplementary Fig 5).
